# Comparative overall survival of CDK4/6 inhibitors plus an aromatase inhibitor in HR+/HER2− metastatic breast cancer in the US real-world setting☆

**DOI:** 10.1016/j.esmoop.2024.104103

**Published:** 2025-01-03

**Authors:** H.S. Rugo, R.M. Layman, F. Lynce, X. Liu, B. Li, L. McRoy, A.B. Cohen, M. Estevez, G. Curigliano, A. Brufsky

**Affiliations:** 1 University of California San Francisco Helen Diller Family Comprehensive Cancer Center, San Francisco, USA; 2 The University of Texas MD Anderson Cancer Center, Houston, USA; 3 Dana-Farber Cancer Institute, Boston, USA; 4 Pfizer Inc., New York, USA; 5 Flatiron Health Inc., New York, USA; 6 NYU Langone School of Medicine, New York, USA; 7 European Institute of Oncology, IRCCS, Milan, Italy; 8 Department of Oncology and Hemato-Oncology, University of Milano, Milan, Italy; 9 UPMC Hillman Cancer Center, University of Pittsburgh Medical Center, Pittsburgh, USA

**Keywords:** comparative effectiveness, cyclin-dependent kinase 4/6 inhibitor, abemaciclib, ribociclib, palbociclib

## Abstract

**Background:**

Randomized controlled trials have shown inconsistent overall survival (OS) benefit among the three cyclin-dependent kinase 4/6 inhibitors (CDK4/6i) as first-line (1L) treatment of patients with hormone receptor-positive (HR+)/human epidermal growth factor receptor 2-negative (HER2−) metastatic breast cancer (mBC). Several real-world studies compared CDK4/6i effectiveness, with inconsistent findings. This study compared overall survival (OS) of patients with HR+/HER2− mBC receiving 1L palbociclib, ribociclib, or abemaciclib, in combination with an aromatase inhibitor (AI), in US clinical practice.

**Patients and methods:**

This retrospective study used real-world data from the Flatiron Health electronic health record-derived deidentified longitudinal database. Patients with HR+/HER2− mBC aged ≥18 years at mBC diagnosis started 1L CDK4/6i therapy (index treatment) between February 2015 and November 2023, with a potential ≥6-month follow-up. OS was defined as months from start of index treatment to death. Stabilized inverse probability of treatment weighting (sIPTW; primary analysis) was used to balance baseline patient characteristics. Multivariable Cox proportional hazards model was carried out as a sensitivity analysis.

**Results:**

Of 9146 eligible patients, 6831, 1279, and 1036 received palbociclib plus AI, ribociclib plus AI, or abemaciclib plus AI, respectively. After sIPTW, baseline characteristics were balanced between treatment groups. After sIPTW, no significant OS differences were found between treatment groups [ribociclib versus palbociclib: adjusted hazard ratio (aHR) 0.98, 95% confidence interval (CI) 0.87-1.10, *P* = 0.7531; abemaciclib versus palbociclib: aHR 0.95, 95% CI 0.84-1.08, *P* = 0.4292; abemaciclib versus ribociclib: aHR 0.97, 95% CI 0.82-1.14, *P* = 0.6956]. Sensitivity analysis including a subanalysis of patients who started index treatment in 2017 or later also showed no significant OS differences between treatment groups.

**Conclusions:**

This large real-world study suggested that there were no significant OS differences between 1L ribociclib, abemaciclib, and palbociclib in combination with an AI for patients with HR+/HER2− mBC. These findings together with other factors such as safety and quality of life are helpful in the selection of CDK4/6i combination therapy for patients with HR+/HER2− mBC.

## Highlights

• OS was compared between the three CDK4/6i in combination with AI in 9146 HR+/HER2− mBC patients in the US real-world setting.
• There were no significant OS differences between 1L palbociclib, ribociclib, and abemaciclib treatment groups.
• OS results remained generally consistent across various sensitivity and subgroup analyses.

## Introduction

The combination of a cyclin-dependent kinase 4/6 inhibitor (CDK4/6i) with endocrine therapy (ET) has become a standard-of-care first-line (1L) treatment for patients with hormone receptor-positive/human epidermal growth factor receptor 2-negative (HR+/HER2−) advanced/metastatic breast cancer (mBC).^1^^,^^2^ Palbociclib was the first CDK4/6i approved in the United States in 2015, followed by ribociclib and abemaciclib in 2017.^3^, ^4^, ^5^ When used in combination with an aromatase inhibitor (AI) as 1L treatment for patients with HR+/HER2− mBC in phase III randomized controlled trials [RCTs; PALOMA-2 (palbociclib), MONARCH-3 (abemaciclib), and MONALEESA-2 (ribociclib)], all three CDK4/6i significantly prolonged the primary endpoint of progression-free survival (PFS) compared with placebo plus AI.^6^, ^7^, ^8^ Although median overall survival (OS; secondary endpoint) was numerically longer in the CDK4/6i arm compared with the placebo arm in each of these trials, only ribociclib combination treatment in the MONALEESA-2 trial demonstrated a statistically significant OS difference.^9^, ^10^, ^11^

In the absence of head-to-head RCTs, several indirect treatment comparison analyses have evaluated the relative effectiveness of the CDK4/6i using data from the PALOMA, MONALEESA, and MONARCH clinical trials.^12^, ^13^, ^14^, ^15^ These studies did not show significant differences in PFS and/or OS between the three CDK4/6i when used in combination with ET. To date, several real-world studies have evaluated the comparative effectiveness of the different CDK4/6i.^16^, ^17^, ^18^, ^19^, ^20^, ^21^, ^22^, ^23^, ^24^, ^25^ Findings were inconsistent across these studies, limited by small sample sizes and short follow-up, although most did not show significant differences in real-world PFS and/or OS among CDK4/6i.

Large-scale, real-world comparative studies with longer follow-up are needed to further elucidate the relative effectiveness of the three approved CDK4/6i in clinical practice. This comparative effectiveness analysis used an US nationwide electronic health record (EHR)-derived database to compare OS of patients with HR+/HER2− mBC receiving 1L palbociclib, ribociclib, or abemaciclib, in combination with an AI, in routine clinical practice.

## Patients and methods

This retrospective database analysis was conducted in accordance with the Guidelines for Good Pharmacoepidemiology Practice, Good Practices for Outcomes Research issued by the International Society for Pharmacoeconomics and Outcomes Research, and Good Practices for Real-world Data Studies of Treatment and/or Comparative Effectiveness. This study is retrospective and noninterventional and uses anonymized data, therefore it is exempt from institutional review board approval and included a waiver of informed consent.

### Study design and data source

P-VERIFY (Palbociclib Verifying Evidence of Real-world Impact Study; ClinicalTrials.gov Identifier: NCT06495164) was a retrospective comparative effectiveness study using the Flatiron Health EHR-derived deidentified longitudinal database. This is a US nationwide database containing patient-level structured and unstructured data, curated using natural language processing with machine learning and technology-enabled abstraction.^26^, ^27^, ^28^ The data were subject to obligations to prevent reidentification and protect patient confidentiality. Study variables were validated using Flatiron Health’s quality and performance assessment frameworks.^29^, ^30^, ^31^ The database includes >721 000 patients with breast cancer, originating from ∼280 cancer clinics (∼800 sites of care). The data model characterizes the entire journey of a patient with breast cancer, from early to metastatic disease, with treatment details, clinical characteristics, and validated real-world outcomes. Lines of therapy in the database were oncologist-defined and rule-based.^32^

### Patients

**Figure 1:**
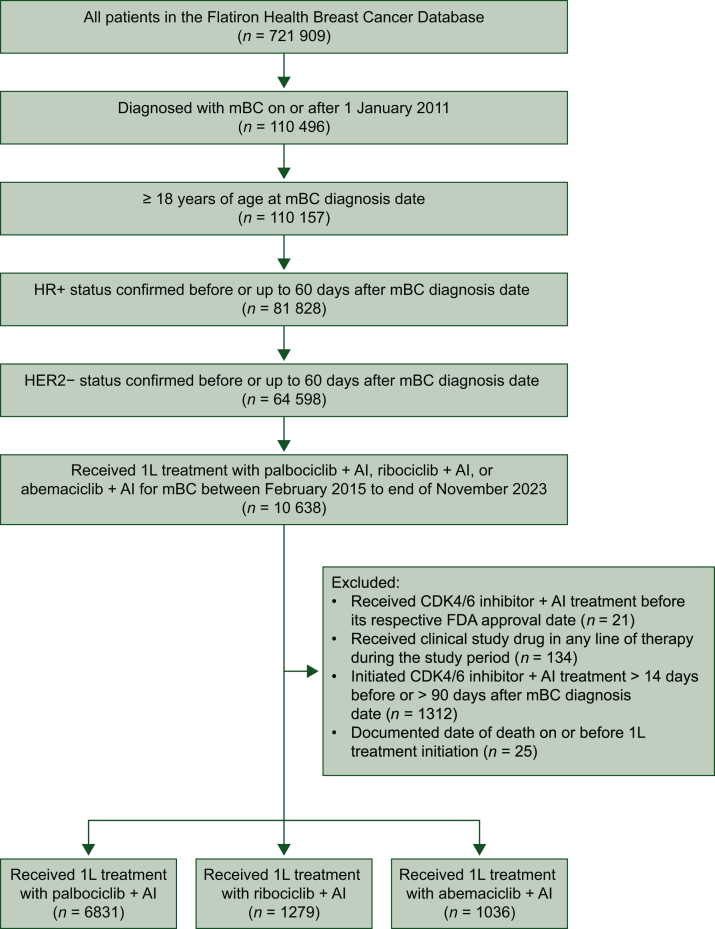
**Flowchart of the study cohort.** 1L, first-line; AI, aromatase inhibitor; CDK4/6, cyclin-dependent kinase 4/6; FDA, Food and Drug Administration; HER2−, human epidermal growth factor receptor 2-negative; HR+, hormone receptor-positive; mBC, metastatic breast cancer.

All eligible patients with HR+/HER2− mBC treated with a CDK4/6i in combination with an AI as 1L therapy in the mBC cohort were included in the current analysis (Figure 1). HR+ was defined as an estrogen receptor (ER)-positive or progesterone receptor (PR)-positive biomarker test any time before or up to 60 days after mBC diagnosis or having documentation of HR+ disease in the absence of ER/PR information. Patients were included if they were ≥18 years of age at mBC diagnosis, started index treatment (palbociclib plus AI, ribociclib plus AI, or abemaciclib plus AI) as 1L therapy within 14 days before or 90 days after mBC diagnosis between February 2015 and November 2023 (index period), and had a potential follow-up of at least 6 months from index treatment to the data cut-off date of 31 May 2024. Patients who participated in clinical trials were excluded. Patients were followed from start of index treatment to the data cut-off date, death, or last medical activity, whichever came first. As all three CDK4/6i were approved and available for treatment in the United States starting in 2017, a subanalysis of patients who started index treatment in 2017 or later was also carried out.

### Outcome

The primary outcome was OS, defined as the number of months from start of index treatment to death.^33^^,^^34^ Date of death was a consensus mortality endpoint based on EHR, Social Security Death Index, and obituary data, validated against the National Death Index.^32^ Patients still alive were censored at the end of the study.

### Baseline variables

Patient demographic and clinical characteristics assessed at baseline included age, sex, race, practice type (academic or community), insurance type, Eastern Cooperative Oncology Group (ECOG) performance status, disease stage at initial diagnosis, visceral metastasis in the lung and/or liver, bone-only metastatic disease, number of disease sites (multiple metastases at the same site were counted as one site), disease-free interval (from initial breast cancer diagnosis to mBC diagnosis), menopausal status at initial diagnosis, and year of index treatment (Table 1).

**Table 1:** Patient characteristics in unadjusted total cohort

Characteristic	Cohort			Standardized difference		
	PAL + AI (*n* = 6831)	RIB + AI (*n* = 1279)	ABE + AI (*n* = 1036)	RIB + AI versus PAL + AI	ABE + AI versus PAL + AI	ABE + AI versus RIB + AI
Age at mBC diagnosis, years						
Mean (SD)	65.6 (11.4)	62.3 (12.8)	63.3 (12.5)	−0.2725	−0.1934	0.0784
Median (IQR)	66.0 (16.0)	64.0 (19.0)	64.0 (18.0)			
Sex, *n* (%)						
Male	75 (1.1)	7 (0.5)	12 (1.2)	−0.0610	0.0057	0.0665
Female	6756 (98.9)	1272 (99.5)	1024 (98.8)			
Race, *n* (%)						
White	4385 (64.2)	759 (59.3)	572 (55.2)	−0.0999	−0.1839	−0.0836
Black	608 (8.9)	125 (9.8)	124 (12.0)	0.0300	0.1005	0.0706
Other	1838 (26.9)	395 (30.9)	340 (32.8)	0.0878	0.1294	0.0415
Practice type, *n* (%)						
Community	5696 (83.4)	1140 (89.1)	902 (87.1)	0.1675	0.1039	−0.0639
Academic	1135 (16.6)	139 (10.9)	134 (12.9)			
Insurance type, *n* (%)						
Commercial health plan plus any other	2515 (36.8)	411 (32.1)	340 (32.8)	−0.0986	−0.0840	0.0146
Commercial health plan	2143 (31.4)	495 (38.7)	401 (38.7)	0.1541	0.1542	0.0001
Medicare	323 (4.7)	44 (3.4)	27 (2.6)	−0.0651	−0.1131	−0.0487
Medicaid	116 (1.7)	34 (2.7)	15 (1.4)	0.0658	−0.0201	−0.0854
Other payer type	1734 (25.4)	295 (23.1)	253 (24.4)	−0.0542	−0.0223	0.0319
Disease stage at initial diagnosis, *n* (%)						
I	719 (10.5)	126 (9.9)	119 (11.5)	−0.0223	0.0307	0.0530
II	1552 (22.7)	308 (24.1)	199 (19.2)	0.0322	−0.0863	−0.1185
III	732 (10.7)	136 (10.6)	108 (10.4)	−0.0027	−0.0095	−0.0068
IV	3452 (50.5)	638 (49.9)	562 (54.2)	−0.0130	0.0744	0.0874
Not documented	376 (5.5)	71 (5.6)	48 (4.6)	0.0021	−0.0397	−0.0418
ECOG PS, *n* (%)						
0	2363 (34.6)	498 (38.9)	414 (40.0)	0.0902	0.1112	0.0210
1	1809 (26.5)	343 (26.8)	266 (25.7)	0.0076	−0.0184	−0.0260
2, 3, or 4	816 (11.9)	127 (9.9)	100 (9.7)	−0.0646	−0.0739	−0.0093
Not documented	1843 (27.0)	311 (24.3)	256 (24.7)	−0.0610	−0.0519	0.0092
Disease-free interval, *n* (%)						
*De novo* mBC	3452 (50.5)	638 (49.9)	562 (54.2)	−0.0130	0.0744	0.0874
≤1 year	265 (3.9)	54 (4.2)	52 (5.0)	0.0174	0.0553	0.0380
>1-5 years	1096 (16.0)	195 (15.2)	157 (15.2)	−0.0220	−0.0245	−0.0026
>5 years	2018 (29.5)	392 (30.6)	265 (25.6)	0.0241	−0.0888	−0.1130
Visceral metastasis, *n* (%)^a^						
No	4513 (66.1)	839 (65.6)	614 (59.3)	−0.0099	−0.1409	−0.1310
Yes	2318 (33.9)	440 (34.4)	422 (40.7)			
Bone-only metastasis, *n* (%)^b^						
No	3608 (52.8)	676 (52.9)	615 (59.4)	0.0007	0.1322	0.1314
Yes	3223 (47.2)	603 (47.1)	421 (40.6)			
Number of metastatic sites, *n* (%)^c^						
1	4050 (59.3)	752 (58.8)	569 (54.9)	−0.0100	−0.0883	−0.0783
2	1537 (22.5)	310 (24.2)	269 (26.0)	0.0411	0.0809	0.0399
≥3	609 (8.9)	113 (8.8)	105 (10.1)	−0.0028	0.0416	0.0444
Not documented	635 (9.3)	104 (8.1)	93 (9.0)	−0.0413	−0.0111	0.0302
Menopausal status at initial diagnosis, *n* (%)						
Premenopausal	1158 (17.0)	364 (28.5)	236 (22.8)	0.2773	0.1465	−0.1304
Postmenopausal	5247 (76.8)	838 (65.5)	724 (69.9)	−0.2512	−0.1572	0.0934
Not documented	351 (5.1)	70 (5.5)	64 (6.2)	0.0149	0.0450	0.0301
Not applicable (patient is male)	75 (1.1)	7 (0.5)	12 (1.2)	−0.0610	0.0057	0.0665
Year of index date, *n* (%)						
2015	441 (6.5)	0	0			
2016	655 (9.6)	0	0			
2017	712 (10.4)	69 (5.4)	0			
2018	789 (11.6)	124 (9.7)	60 (5.8)			
2019	870 (12.7)	107 (8.4)	120 (11.6)			
2020	922 (13.5)	100 (7.8)	147 (14.2)			
2021	1027 (15.0)	85 (6.6)	196 (18.9)			
2022	872 (12.8)	232 (18.1)	239 (23.1)			
2023	543 (7.9)	562 (43.9)	274 (26.4)			
Median follow-up duration (IQR), months	33.0 (34.8)	16.2 (22.5)	21.4 (25.0)			

ABE, abemaciclib; AI, aromatase inhibitor; ECOG PS, Eastern Cooperative Oncology Group performance status; IQR, interquartile range; mBC, metastatic breast cancer; PAL, palbociclib; RIB, ribociclib; SD, standard deviation.

a Visceral disease is defined as metastatic disease in the lung and/or liver; patients could have had other sites of metastases.

b Bone-only disease is defined as metastatic disease in the bone only.

c Multiple metastases at the same site were counted as one site (e.g. three bone metastases in the spine was considered only one site).

### Statistical analysis

Descriptive analyses were conducted to describe patient baseline characteristics. Continuous variables were summarized with means, standard deviations, medians, and interquartile ranges. Categorical variables were reported as counts and percentages. OS was estimated using the Kaplan–Meier method and displayed graphically. The Cox proportional hazards model was used to compute hazard ratios and corresponding 95% confidence intervals (CIs). Three methods were used for comparative analysis: (i) an unadjusted analysis without adjusting for baseline patient characteristics; (ii) the stabilized inverse probability of treatment weighting (sIPTW) method (primary analysis); and (iii) multivariable Cox proportional hazards model (sensitivity analysis). The sIPTW method was employed to balance baseline demographic and clinical characteristics between treatment groups. A multivariable multinomial logistic regression model was used to compute propensity scores (i.e. treatment probabilities). Variables used in the estimation of propensity scores were age, sex, race, practice type, ECOG performance status, disease stage at initial diagnosis, visceral metastasis, bone-only disease, number of disease sites, and disease-free interval (from initial breast cancer diagnosis to mBC diagnosis). The balance in these baseline characteristics was assessed using a standardized mean differences approach, with values ≥ 0.1 indicating a non-negligible imbalance. The same variables used in the estimation of propensity scores were included in the multivariable Cox proportional hazards model as covariates. All data analyses were conducted with SAS version 9.4 or higher.

## Results

### Patients

Of the 9146 patients who were eligible for this analysis, 6831, 1279, and 1036 patients received treatment with palbociclib plus AI, ribociclib plus AI, and abemaciclib plus AI, respectively (Figure 1). The proportions of ER+ and PR+ were almost the same among the three groups: palbociclib plus AI (99.4% and 85.4%); ribociclib plus AI (99.5% and 86.0%); and abemaciclib plus AI (99.1% and 86.1%). Of the three patient groups, patients in the palbociclib group were 2 years older (based on median age) than patients in the abemaciclib and ribociclib groups (Table 1). The ribociclib group had the highest proportion of premenopausal patients (28.5% versus 17.0% and 22.8% in the palbociclib and abemaciclib groups, respectively), and the palbociclib group had the lowest proportion of patients with an ECOG performance status of 0 (34.6% versus 38.9% and 40.0% in the ribociclib and abemaciclib groups, respectively). The abemaciclib group had the highest proportion of patients with visceral metastasis (40.7% versus 33.9% and 34.4% in palbociclib and ribociclib groups, respectively) and the lowest proportion of patients with bone-only metastasis (40.6% versus 47.2% and 47.1%). After sIPTW, baseline demographics and clinical characteristics were generally balanced between the palbociclib, ribociclib, and abemaciclib groups (Table 2). Median duration of follow-up before and after sIPTW remained unchanged at 33 months for patients treated with palbociclib plus AI, ∼16 months for patients treated with ribociclib plus AI, and ∼21 months for patients treated with abemaciclib plus AI (Tables 1 and 2).

**Table 2:** Patient characteristics after sIPTW

Characteristic	Cohort			Standardized difference		
	PAL + AI (*n* = 6832)	RIB + AI (*n* = 1274)	ABE + AI (*n* = 1038)	RIB + AI versus PAL + AI	ABE + AI versus PAL + AI	ABE + AI versus RIB + AI
Age at mBC diagnosis, years						
Mean (SD)	65.0 (11.7)	64.6 (12.0)	64.7 (12.1)	−0.0297	−0.0198	0.0097
Median (IQR)	66.0 (17.0)	66.0 (17.0)	66.0 (17.0)			
Sex, *n* (%)						
Male	70 (1.0)	11 (0.9)	11 (1.1)	−0.0178	0.0068	0.0245
Female	6762 (99.0)	1263 (99.1)	1026 (98.9)			
Race, *n* (%)						
White	4272 (62.5)	797 (62.6)	654 (63.0)	0.0008	0.0093	0.0086
Black	638 (9.3)	117 (9.2)	94 (9.1)	−0.0063	−0.0081	−0.0018
Other	1922 (28.1)	360 (28.3)	290 (27.9)	0.0032	−0.0048	−0.0081
Practice type, *n* (%)						
Community	5782 (84.6)	1085 (85.2)	872 (84.0)	0.0154	−0.0169	−0.0323
Academic	1050 (15.4)	189 (14.8)	166 (16.0)			
Insurance type, *n* (%)						
Commercial health plan plus any other	2466 (36.1)	433 (34.0)	355 (34.2)	−0.0434	−0.0399	0.0034
Commercial health plan	2167 (31.7)	472 (37.1)	392 (37.8)	0.1128	0.1281	0.0152
Medicare	310 (4.5)	50 (3.9)	30 (2.8)	−0.0292	−0.0898	−0.0610
Medicaid	121 (1.8)	32 (2.5)	13 (1.3)	0.0528	−0.0387	−0.0907
Other payer type	1768 (25.9)	286 (22.4)	248 (23.9)	−0.0809	−0.0466	0.0343
Disease stage at initial diagnosis, *n* (%)						
I	720 (10.5)	136 (10.7)	112 (10.8)	0.0052	0.0091	0.0039
II	1538 (22.5)	287 (22.5)	238 (22.9)	0.0005	0.0092	0.0088
III	731 (10.7)	136 (10.7)	110 (10.6)	−0.0003	−0.0044	−0.0041
IV	3473 (50.8)	645 (50.6)	523 (50.4)	−0.0046	−0.0096	−0.0050
Not documented	370 (5.4)	70 (5.5)	56 (5.4)	0.0027	−0.0023	−0.0051
ECOG PS, *n* (%)						
0	2444 (35.8)	457 (35.9)	369 (35.5)	0.0025	−0.0050	−0.0075
1	1806 (26.4)	329 (25.9)	275 (26.5)	−0.0133	0.0008	0.0141
2, 3, or 4	780 (11.4)	147 (11.5)	119 (11.5)	0.0032	0.0018	−0.0015
Not documented	1801 (26.4)	341 (26.7)	275 (26.5)	0.0082	0.0035	−0.0048
Disease-free interval, *n* (%)						
*De novo* mBC	3473 (50.8)	645 (50.6)	523 (50.4)	−0.0046	−0.0096	−0.0050
≤1 year	276 (4.0)	52 (4.1)	42 (4.1)	0.0014	0.0007	−0.0007
>1-5 years	1082 (15.8)	202 (15.8)	162 (15.6)	0.0000	−0.0065	−0.0065
>5 years	2001 (29.3)	376 (29.5)	311 (30.0)	0.0044	0.0154	0.0109
Visceral metastasis, *n* (%)^a^						
No	4458 (65.3)	831 (65.2)	680 (65.5)	−0.0006	0.0060	0.0066
Yes	2374 (34.7)	443 (34.8)	358 (34.5)			
Bone-only metastasis, *n* (%)^b^						
No	3661 (53.6)	683 (53.6)	557 (53.7)	0.0004	0.0020	0.0016
Yes	3171 (46.4)	591 (46.4)	481 (46.3)			
Number of metastatic sites, *n* (%)^c^						
1	4010 (58.7)	745 (58.5)	611 (58.8)	−0.0040	0.0029	0.0069
2	1585 (23.2)	304 (23.9)	240 (23.1)	0.0158	−0.0025	−0.0183
≥3	615 (9.0)	111 (8.7)	92 (8.9)	−0.0112	−0.0036	0.0076
Not documented	622 (9.1)	114 (8.9)	95 (9.2)	−0.0055	0.0023	0.0078
Menopausal status at initial diagnosis, *n* (%)						
Premenopausal	1265 (18.5)	279 (21.9)	204 (19.6)	0.0849	0.0279	−0.0570
Postmenopausal	5138 (75.2)	919 (72.1)	762 (73.4)	−0.0702	−0.0415	0.0286
Not documented	359 (5.2)	65 (5.1)	61 (5.9)	−0.0066	0.0283	0.0349
Not applicable (patient is male)	70 (1.0)	11 (0.9)	11 (1.1)	−0.0178	0.0068	0.0245
Year of index date, *n* (%)						
2015	440 (6.4)	0	0			
2016	657 (9.6)	0	0			
2017	713 (10.4)	70 (5.5)	0			
2018	789 (11.6)	122 (9.6)	58 (5.6)			
2019	870 (12.7)	102 (8.0)	125 (12.0)			
2020	928 (13.6)	92 (7.2)	146 (14.0)			
2021	1026 (15.0)	80 (6.3)	189 (18.2)			
2022	870 (12.7)	226 (17.7)	247 (23.8)			
2023	539 (7.9)	582 (45.7)	273 (26.3)			
Median follow-up duration (IQR), months	33.0 (34.7)	15.7 (20.8)	21.5 (25.0)			

ABE, abemaciclib; AI, aromatase inhibitor; ECOG PS, Eastern Cooperative Oncology Group performance status; IQR, interquartile range; mBC, metastatic breast cancer; PAL, palbociclib; RIB, ribociclib; sIPTW, stabilized inverse probability of treatment weighting; SD, standard deviation.

a Visceral disease is defined as metastatic disease in the lung and/or liver; patients could have had other sites of metastases.

b Bone-only disease is defined as metastatic disease in the bone only.

c Multiple metastases at the same site were counted as one site (e.g. three bone metastases in the spine was considered only one site).

### Overall survival

Among 9146 patients in the unadjusted analysis, a total of 3714 deaths were observed: 3096 of 6831 (45.3%) in the palbociclib group, 328 of 1279 (25.6%) in the ribociclib group, and 290 of 1036 (28.0%) in the abemaciclib group (Supplementary Table S1, available at https://doi.org/10.1016/j.esmoop.2024.104103). A total of 5432 patients were censored: 3735 of 6831 (54.7%) in the palbociclib group, 951 of 1279 (74.4%) in the ribociclib group, and 746 of 1036 (72.0%) in the abemaciclib group.

**Figure 2:**
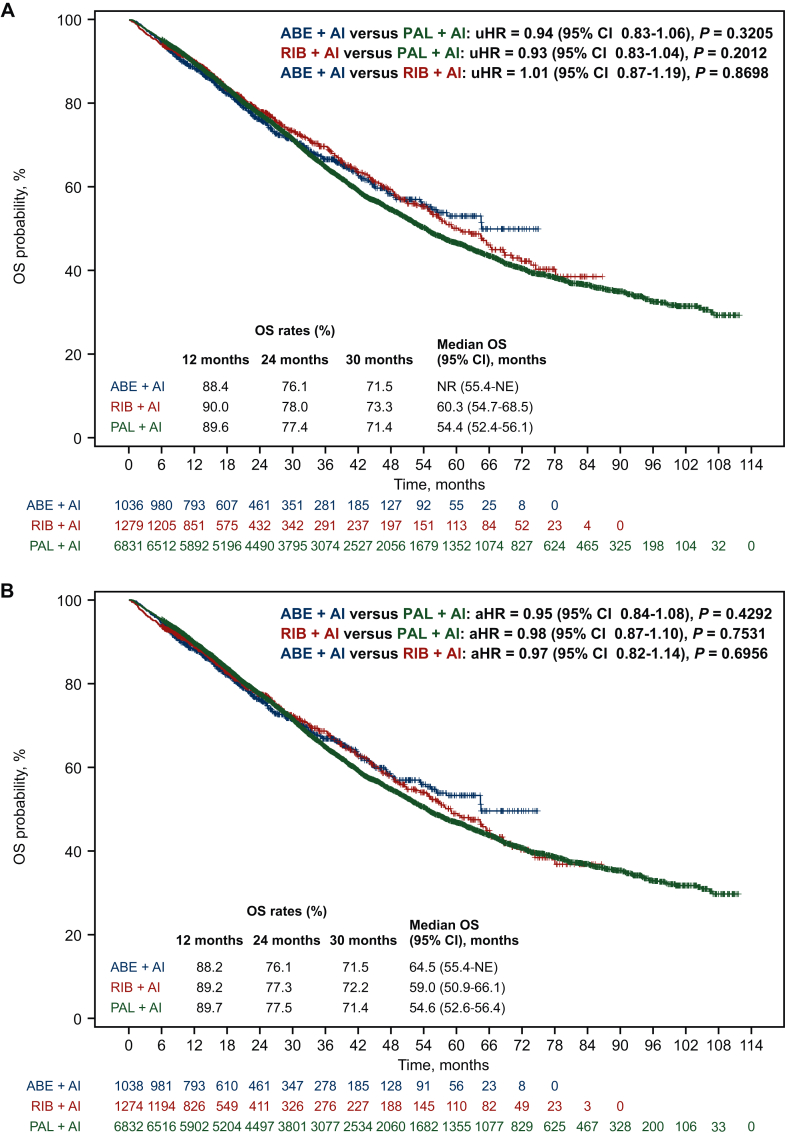
**Overall survival in the (A) unadjusted analysis and (B) after sIPTW among the three CDK4/6 inhibitors.** ABE, abemaciclib; aHR, adjusted hazard ratio; AI, aromatase inhibitor; CDK4/6, cyclin-dependent kinase 4/6; CI, confidence interval; HR, hazard ratio; NE, not estimable; NR, not reached; OS, overall survival; PAL, palbociclib; RIB, ribociclib; sIPTW, stabilized inverse probability of treatment weighting; uHR, unadjusted hazard ratio.

**Figure 3:**
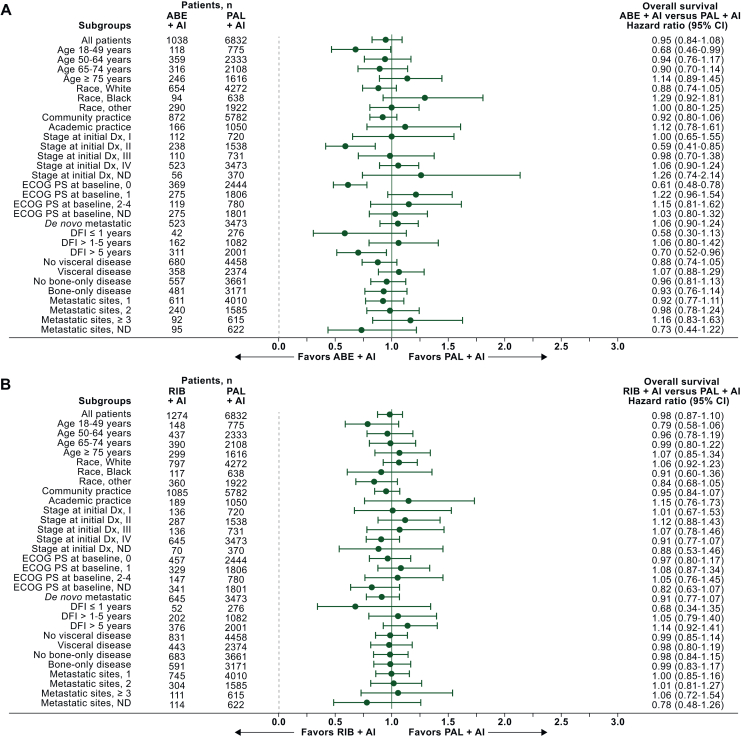

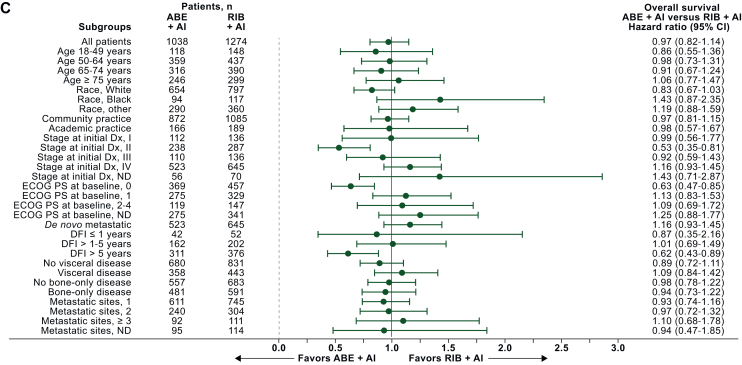
**Forest plot of overall survival by subgroup after sIPTW: (A) abemaciclib + AI versus palbociclib + AI, (B) ribociclib + AI versus palbociclib + AI, and (C) abemaciclib + AI versus ribociclib + AI.** ABE, abemaciclib; AI, aromatase inhibitor; CI, confidence interval; DFI, disease-free interval; Dx, diagnosis; ECOG PS, Eastern Cooperative Oncology Group performance status ND, not determined; PAL, palbociclib; RIB, ribociclib; sIPTW, stabilized inverse probability of treatment weighting.

There were no significant differences in OS between treatment groups in the unadjusted analysis, with an unadjusted hazard ratio of 0.93 (95% CI 0.83-1.04, *P* = 0.2012), 0.94 (95% CI 0.83-1.06, *P* = 0.3205), and 1.01 (95% CI 0.87-1.19, *P* = 0.8698) for ribociclib versus palbociclib, abemaciclib versus palbociclib, and abemaciclib versus ribociclib pairwise group comparisons, respectively (Figure 2A). In the adjusted analysis, no significant differences in OS were found between treatment groups after sIPTW (Figure 2B). The adjusted hazard ratio (aHR) was 0.98 (95% CI 0.87-1.10, *P* = 0.7531) for the ribociclib versus palbociclib, 0.95 (95% CI 0.84-1.08, *P* = 0.4292) for the abemaciclib versus palbociclib, and 0.97 (95% CI 0.82-1.14, *P* = 0.6956) for the abemaciclib versus ribociclib. After sIPTW, 12-, 24-, and 30-month OS rates in the treatment groups were: palbociclib, 89.7%, 77.5%, and 71.4%; ribociclib, 89.2%, 77.3%, and 72.2%; abemaciclib, 88.2%, 76.1%, and 71.5%. After sIPTW, median OS was 54.6 months (95% CI 52.6-56.4 months) in the palbociclib group, 59.0 months (95% CI 50.9-66.1 months) in the ribociclib group, and 64.5 months (95% CI 55.4 months-not estimable) in the abemaciclib group. Results from the subgroup analysis of OS after sIPTW in each of the pairwise group comparisons were generally consistent with results from the overall cohort (Figure 3).

A sensitivity analysis using a multivariable Cox proportional hazards model showed no significant differences in OS between treatment groups. The aHR was 0.94 (95% CI 0.84-1.06, *P* = 0.3216) when comparing the ribociclib group versus the palbociclib group, 0.94 (95% CI 0.84-1.07, *P* = 0.3603) when comparing the abemaciclib group versus the palbociclib group, and 1.00 (95% CI 0.85-1.17, *P* = 0.9851) when comparing the abemaciclib group versus the ribociclib group.

### Subanalysis of patients who started index treatment in 2017 or later

This analysis was carried out to include only patients who initiated treatment when all three CDK4/6i were available for use. This subanalysis included 5735, 1279, and 1036 patients treated with palbociclib plus AI, ribociclib plus AI, and abemaciclib plus AI, respectively (Supplementary Table S2, available at https://doi.org/10.1016/j.esmoop.2024.104103). After sIPTW, baseline demographics and clinical characteristics were generally balanced between treatment groups (Supplementary Table S3, available at https://doi.org/10.1016/j.esmoop.2024.104103).

Consistent with results in the overall study cohort, the pairwise comparisons for patients who initiated treatment after 2017 did not demonstrate significant differences in OS in the unadjusted analysis (Supplementary Figure S1A, available at https://doi.org/10.1016/j.esmoop.2024.104103) or after sIPTW (Supplementary Figure S1B, available at https://doi.org/10.1016/j.esmoop.2024.104103). After sIPTW, the aHR was 1.00 (95% CI 0.89-1.13, *P* = 0.9728) for the ribociclib versus palbociclib comparison, 0.96 (95% CI 0.84-1.09, *P* = 0.5326) for the abemaciclib versus palbociclib comparison, and 0.96 (95% CI 0.81-1.13, *P* = 0.6077) for the abemaciclib versus ribociclib comparison (Supplementary Figure S1B, available at https://doi.org/10.1016/j.esmoop.2024.104103). Results from the subgroup analysis of OS after sIPTW in each of the pairwise group comparisons were generally consistent with results from the overall cohort of patients who started treatment in 2017 or later (Supplementary Figure S2, available at https://doi.org/10.1016/j.esmoop.2024.104103).

Multivariable Cox proportional hazards model also showed no significant differences in OS between treatment groups in patients who started treatment in 2017 or later. Specifically, the aHR was 0.96 (95% CI 0.86-1.08, *P* = 0.5131) for the ribociclib versus palbociclib comparison, 0.96 (95% CI 0.85-1.09, *P* = 0.5340) for the abemaciclib versus palbociclib comparison, and 1.00 (95% CI 0.85-1.17, *P* = 0.9997) for the abemaciclib versus ribociclib comparison. Similar results were found when using 2018 or later as index treatment date.

## Discussion

The importance of real-world evidence has been increasingly recognized as it serves to expand upon and complement data from RCTs to inform clinical decision making.^35^ In this article, we report results from the largest real-world study to date comparing OS in patients receiving palbociclib, ribociclib, or abemaciclib, in combination with an AI, as 1L treatment for HR+/HER2− mBC. Overall, we found no significant differences when comparing OS between different CDK4/6i treatment groups (after sIPTW, all aHR = 0.95-1.00; *P* > 0.05).

Across phase III trials, all three CDK4/6i, in combination with an AI, numerically prolonged median OS compared with placebo plus an AI when used as 1L treatment for patients with HR+/HER2− mBC.^9^, ^10^, ^11^ Median OS in the CDK4/6i arm versus the placebo arm was 53.9 versus 51.2 months, respectively, in the PALOMA-2 trial evaluating palbociclib plus an AI (hazard ratio 0.96, 95% CI 0.78-1.18, *P* = 0.34)^10^; and 66.8 versus 53.7 months, respectively, in the MONARCH-3 trial evaluating abemaciclib plus an AI (hazard ratio 0.804, 95% CI 0.637-1.015, *P* = 0.0664).^9^ Only ribociclib plus an AI demonstrated a statistically significant OS difference, with a median of 63.9 months versus 51.4 months in the placebo arm (hazard ratio 0.76, 95% CI 0.63-0.93), *P* = 0.008) in the MONALEESA-2 trial.^11^ Of note, in the extended follow-up of the PARSIFAL trial,^36^ PARSIFAL-LONG, patients treated with palbociclib in combination with either fulvestrant or letrozole had a median OS of 65.4 months,^37^ which is similar to the OS benefit observed with abemaciclib and ribociclib in MONARCH-3 and MONALEESA-2, respectively. Differences in OS benefit between trials may be due to several factors, including study design elements such as randomization scheme, sample sizes, loss to follow-up or missing data on survival outcomes, subsequent treatments, and differences in patient characteristics.^38^^,^^39^ Where long survival post-index treatment occurs, the impact of subsequent therapies and loss to follow-up may interfere with the ability to demonstrate survival differences when they exist.^39^^,^^40^ Consistent with the findings of our study, several indirect treatment comparison studies evaluating CDK4/6i plus AI data across RCTs did not find significant OS differences between the different CDK4/6i.^12^, ^13^, ^14^, ^15^

Although RCTs are the gold standard for evaluating treatment efficacy, the generalizability of their results to routine care can be limited due to relatively small sample sizes and stringent enrollment criteria.^41^^,^^42^ Real-world studies in larger and more diverse patient populations are needed to better understand treatment effectiveness and inform decision making in routine clinical practice.^42^^,^^43^ A growing body of real-world evidence has demonstrated a significant OS benefit of 1L palbociclib plus ET versus ET alone in patients with HR+/HER2− mBC, with findings remaining consistent across diverse patient subsets, including those who were elderly, African American, or had metastases of the lung or liver.^33^^,^^34^^,^^44^, ^45^, ^46^

Similar to our study, several prior real-world studies evaluated the comparative effectiveness of CDK4/6i when used in combination with ET to treat patients with HR+/HER2− mBC.^16^, ^17^, ^18^, ^19^, ^20^, ^21^, ^22^, ^23^, ^24^, ^25^ However, to our knowledge, this study is the first to compare these treatments in the United States and includes representation of all eligible patients in a large, commercially available oncology dataset. This study with a sample size of nearly 10 000 patients is the largest study of this type conducted to date. Our findings are consistent with most prior studies, showing no significant OS differences between treatment groups receiving different CDK4/6i.^16^^,^^17^^,^^20^^,^^22^, ^23^, ^24^, ^25^ One exception was a retrospective analysis of hospital records in the UK, which showed a significant OS difference between the three different CDK4/6i treatment groups in an unadjusted analysis, with the longest OS observed in the palbociclib plus ET group.^21^ Additionally, a few studies either did not report comparative OS results (e.g. a multicenter, real-world, Italian study, PALMARES-2)^19^ or did not carry out tests for statistical significance.^18^

It is important to note the significance of the hazard ratio in a time-to-event comparative analysis versus the median OS. Hazard ratio is the statistics used by the Food and Drug Administration for regulatory evaluation of relative efficacy between treatments. Median OS is only a descriptive statistic about the survival probability at a given time point. If the majority of patients are censored due to short follow-up, the median OS is neither stable nor reliable.^47^^,^^48^ In the current study, the median OS in the abemaciclib and ribociclib cohorts may not be stable given the few remaining patients at risk at time points beyond ∼30 months, with only 26.8% and 21.7% of patients, respectively, at risk at 36 months. In this context, landmark OS rates may offer a more appropriate or reliable measure,^48^ especially considering the relatively small sample sizes and short follow-up times in the ribociclib and abemaciclib groups in our study. Thus, the similar 12-, 24-, and 30-month OS rates reported across CDK4/6i groups, in conjunction with the estimated aHR = 0.95-1.00 after sIPTW (*P* > 0.05), further add to the robustness of our study findings.

Strengths of our analysis include the diversity of patients represented and the comprehensiveness of data collected in a US nationwide, longitudinal database. In this database, EHR-derived data were validated using quality and performance assessment frameworks,^29^, ^30^, ^31^ and date of death was a consensus mortality endpoint based on multiple sources and validated against the gold-standard National Death Index.^32^^,^^49^ Our study had a large sample size (*N* = 9146), representing the largest real-world study conducted to date evaluating the comparative effectiveness of CDK4/6i. The consistency of findings across different comparative methods, including the unadjusted analysis, the primary analysis after sIPTW, and the sensitivity analysis using a multivariable regression model, contributed to the study’s internal validity. Similarly, findings remained consistent in the subanalysis of patients who started treatment in 2017 or later, when all CDK4/6i were commercially available in the United States.

Our study has several limitations. This was a retrospective database analysis, with the potential for treatment selection bias and inaccurate or incomplete data capture. No causal relationship could be made. The sample sizes in the ribociclib and abemaciclib groups (*n* = 1279 and *n* = 1036, respectively) were small relative to the palbociclib group (*n* = 6831), precluding a formal, powered, noninferiority analysis. The statistical non-significant differences in OS between the three CDK4/6i in the current analysis do not demonstrate equivalence; a formal noninferiority or equivalence analysis would be needed to draw such conclusions. In addition, some of the subgroups may be limited by an insufficient sample size. Median follow-up times in the ribociclib and abemaciclib groups (16.2 and 21.4 months, respectively) were also short relative to the palbociclib group (33.0 months). Due to the small number of patients at risk after month 30 in the ribociclib and abemaciclib cohorts, point estimates of OS beyond this time point, including the median, were not stable.^47^^,^^48^ Although sIPTW and multivariable analyses were used to adjust for baseline characteristics between treatment groups, these methodologies cannot account for potential unmeasured confounders, such as endocrine sensitivity, adjuvant therapies, and subsequent treatments. This study did not report real-world PFS because the information was not available on the cohort of interest at the time of the study; also, disease progression in EHR is not assessed based on standard criteria such as RECIST.^33^^,^^34^ OS was selected as primary endpoint in this study because OS among the RCTs for the CDK4/6i class has differed, additional evidence can aid in clinical decision making, and the OS variable has been well validated by Flatiron Health.^32^ Finally, results may not be generalizable to patient populations that were not represented in the Flatiron Health database.

### Conclusion

This study represents the largest real-world comparative analysis of OS between CDK4/6i in combination with AI conducted to date. Although further research is needed, our findings suggest that there are no significant OS differences between 1L ribociclib, abemaciclib, or palbociclib used in combination with an AI for patients with HR+/HER2− mBC in routine clinical practice in the United States. Given that there are no head-to-head RCTs between the three CDK4/6i, the findings of the current comparative OS study together with efficacy and safety profiles from the three CDK4/6i clinical trials can be helpful when choosing an appropriate CDK4/6i for patients with HR+/HER2− mBC.
