# Supplementary material for: Comparative overall survival of CDK4/6 inhibitors plus an aromatase inhibitor in HR+/HER2− metastatic breast cancer in the US real-world setting
Source: ESMO Open. 2025 Jan 3;10(1):104103. doi: 10.1016/j.esmoop.2024.104103 (PMC11758200; doi:10.1016/j.esmoop.2024.104103)
Supplement: Supplementary data [file mmc1.docx]

**Supplement**

Supplementary Table S1. Summary of events in Kaplan–Meier analysis of overall survival

Supplementary Table S2. Patient characteristics in unadjusted cohort of patients who started index treatment from 2017

Supplementary Table S3. Patient characteristics after sIPTW of patients who started index treatment from 2017

Supplementary Figure S1. Overall survival in the (A) unadjusted analysis and (B) after sIPTW among the 3 CDK4/6 inhibitors in patients who started index treatment from 2017

Supplementary Figure S2. Forest plot of overall survival by subgroup after sIPTW in patients who started index treatment from 2017: (A) abemaciclib + AI vs palbociclib + AI, (B) ribociclib + AI vs palbociclib + AI, and (C) abemaciclib + AI vs ribociclib + AI

Supplementary Table S1. Summary of events in Kaplan–Meier analysis of overall survival

| **Cohort** | **Total, *N*** | **Died** | | **Censored** | | **Median follow-up (IQR), months** |
| --- | --- | --- | --- | --- | --- | --- |
|  |  | ***n*** | **%** | ***n*** | **%** |  |
| PAL + AI | 6831 | 3096 | 45.3 | 3735 | 54.7 | 33.0 (34.8) |
| RIB + AI | 1279 | 328 | 25.6 | 951 | 74.4 | 16.2 (22.5) |
| ABE + AI | 1036 | 290 | 28.0 | 746 | 72.0 | 21.4 (25.0) |
| Total | 9146 | 3714 | 40.6 | 5432 | 59.4 | — |

Abbreviations: ABE, abemaciclib; AI, aromatase inhibitor; IQR, interquartile range; PAL, palbociclib; RIB, ribociclib.

Supplementary Table S2. Patient characteristics in unadjusted cohort of patients who started index treatment from 2017

| **Characteristic** | **Cohort** | | | **Standardized difference** | | |
| --- | --- | --- | --- | --- | --- | --- |
|  | **PAL + AI  (n = 5735)** | **RIB + AI  (n = 1279)** | **ABE + AI  (n = 1036)** | **RIB + AI vs PAL + AI** | **ABE + AI vs**  **PAL + AI** | **ABE + AI vs RIB + AI** |
| Age at mBC diagnosis, years | | | | | | |
| Mean (SD) | 66.0 (11.5) | 62.3 (12.8) | 63.3 (12.5) | -0.3006 | -0.2220 | 0.0784 |
| Median (IQR) | 67.0 (16.0) | 64.0 (19.0) | 64.0 (18.0) |  |  |  |
| Sex, *n* (%) | | | | | | |
| Male | 70 (1.2) | 7 (0.5) | 12 (1.2) | -0.0720 | -0.0057 | 0.0665 |
| Female | 5665 (98.8) | 1272 (99.5) | 1024 (98.8) |  |  |  |
| Race, *n* (%) | | | | | | |
| White | 3625 (63.2) | 759 (59.3) | 572 (55.2) | -0.0794 | -0.1632 | -0.0836 |
| Black | 528 (9.2) | 125 (9.8) | 124 (12.0) | 0.0193 | 0.0899 | 0.0706 |
| Other | 1582 (27.6) | 395 (30.9) | 340 (32.8) | 0.0726 | 0.1142 | 0.0415 |
| Practice type, *n* (%) | | | | | | |
| Community | 4797 (83.6) | 1140 (89.1) | 902 (87.1) | 0.1606 | 0.0969 | -0.0639 |
| Academic | 938 (16.4) | 139 (10.9) | 134 (12.9) |  |  |  |
| Insurance type, *n* (%) | | | | | | |
| Commercial health plan plus any other | 2135 (37.2) | 411 (32.1) | 340 (32.8) | -0.1072 | -0.0925 | 0.0146 |
| Commercial health plan | 1864 (32.5) | 495 (38.7) | 401 (38.7) | 0.1298 | 0.1298 | 0.0001 |
| Medicare | 264 (4.6) | 44 (3.4) | 27 (2.6) | -0.0592 | -0.1073 | -0.0487 |
| Medicaid | 106 (1.8) | 34 (2.7) | 15 (1.4) | 0.0546 | -0.0315 | -0.0854 |
| Other payer type | 1366 (23.8) | 295 (23.1) | 253 (24.4) | -0.0178 | 0.0141 | 0.0319 |
| Disease stage at initial diagnosis, *n* (%) | | | | | | |
| I | 609 (10.6) | 126 (9.9) | 119 (11.5) | -0.0253 | 0.0277 | 0.0530 |
| II | 1301 (22.7) | 308 (24.1) | 199 (19.2) | 0.0330 | -0.0855 | -0.1185 |
| III | 588 (10.3) | 136 (10.6) | 108 (10.4) | 0.0124 | 0.0056 | -0.0068 |
| IV | 2914 (50.8) | 638 (49.9) | 562 (54.2) | -0.0186 | 0.0689 | 0.0874 |
| Not documented | 323 (5.6) | 71 (5.6) | 48 (4.6) | -0.0035 | -0.0453 | -0.0418 |
| ECOG PS, *n* (%) | | | | | | |
| 0 | 2070 (36.1) | 498 (38.9) | 414 (40.0) | 0.0587 | 0.0797 | 0.0210 |
| 1 | 1543 (26.9) | 343 (26.8) | 266 (25.7) | -0.0020 | -0.0279 | -0.0260 |
| 2, 3, or 4 | 732 (12.8) | 127 (9.9) | 100 (9.7) | -0.0894 | -0.0987 | -0.0093 |
| Not documented | 1390 (24.2) | 311 (24.3) | 256 (24.7) | 0.0018 | 0.0110 | 0.0092 |
| Disease-free interval, *n* (%) | | | | | | |
| De novo mBC | 2914 (50.8) | 638 (49.9) | 562 (54.2) | -0.0186 | 0.0689 | 0.0874 |
| ≤ 1 year | 230 (4.0) | 54 (4.2) | 52 (5.0) | 0.0107 | 0.0486 | 0.0380 |
| > 1-5 years | 875 (15.3) | 195 (15.2) | 157 (15.2) | -0.0003 | -0.0029 | -0.0026 |
| > 5 years | 1716 (29.9) | 392 (30.6) | 265 (25.6) | 0.0158 | -0.0971 | -0.1130 |
| Visceral metastasis, *n* (%)^a^ | | | | | | |
| No | 3794 (66.2) | 839 (65.6) | 614 (59.3) | -0.0117 | -0.1428 | -0.1310 |
| Yes | 1941 (33.8) | 440 (34.4) | 422 (40.7) |  |  |  |
| Bone-only metastasis, *n* (%)^b^ | | | | | | |
| No | 3042 (53.0) | 676 (52.9) | 615 (59.4) | -0.0038 | 0.1276 | 0.1314 |
| Yes | 2693 (47.0) | 603 (47.1) | 421 (40.6) |  |  |  |
| Number of metastatic sites, *n* (%)^c^ | | | | | | |
| 1 | 3400 (59.3) | 752 (58.8) | 569 (54.9) | -0.0099 | -0.0882 | -0.0783 |
| 2 | 1289 (22.5) | 310 (24.2) | 269 (26.0) | 0.0416 | 0.0815 | 0.0399 |
| ≥ 3 | 512 (8.9) | 113 (8.8) | 105 (10.1) | -0.0033 | 0.0411 | 0.0444 |
| Not documented | 534 (9.3) | 104 (8.1) | 93 (9.0) | -0.0418 | -0.0116 | 0.0302 |
| Menopausal status at initial diagnosis, *n* (%) | | | | | | |
| Premenopausal | 959 (16.7) | 364 (28.5) | 236 (22.8) | 0.2835 | 0.1526 | -0.1304 |
| Postmenopausal | 4418 (77.0) | 838 (65.5) | 724 (69.9) | -0.2566 | -0.1625 | 0.0934 |
| Not documented | 288 (5.0) | 70 (5.5) | 64 (6.2) | 0.0202 | 0.0503 | 0.0301 |
| Not applicable (patient is male) | 70 (1.2) | 7 (0.5) | 12 (1.2) | -0.0720 | -0.0057 | 0.0665 |
| Year of index date, *n* (%) | | | | | | |
| 2017 | 712 (12.4) | 69 (5.4) | 0 | -0.2484 | -0.5324 | -0.3377 |
| 2018 | 789 (13.8) | 124 (9.7) | 60 (5.8) | -0.1265 | -0.2707 | -0.1464 |
| 2019 | 870 (15.2) | 107 (8.4) | 120 (11.6) | -0.2123 | -0.1055 | 0.1075 |
| 2020 | 922 (16.1) | 100 (7.8) | 147 (14.2) | -0.2567 | -0.0527 | 0.2046 |
| 2021 | 1027 (17.9) | 85 (6.6) | 196 (18.9) | -0.3483 | 0.0261 | 0.3739 |
| 2022 | 872 (15.2) | 232 (18.1) | 239 (23.1) | 0.0788 | 0.2009 | 0.1221 |
| 2023 | 543 (9.5) | 562 (43.9) | 274 (26.4) | 0.8460 | 0.4536 | -0.3726 |
| Median follow-up duration (IQR), months | 31.3 (31.0) | 16.2 (22.5) | 21.4 (25.0) |  |  |  |

^a^ Visceral disease is defined as metastatic disease in the lung and/or liver; patients could have had other sites of metastases.
^b^ Bone-only disease is defined as metastatic disease in the bone only.
^c^ Multiple metastases at the same site were counted as one site (eg, 3 bone metastases in the spine was considered only one site).

Abbreviations: ABE, abemaciclib; AI, aromatase inhibitor; ECOG PS, Eastern Cooperative Oncology Group performance status; IQR, interquartile range; mBC, metastatic breast cancer; PAL, palbociclib; RIB, ribociclib; SD, standard deviation.

Supplementary Table S3. Patient characteristics after sIPTW of patients who started index treatment from 2017

| **Characteristic** | **Cohort** | | | **Standardized difference** | | |
| --- | --- | --- | --- | --- | --- | --- |
|  | **PAL + AI  (n = 5737)** | **RIB + AI  (n = 1273)** | **ABE + AI  (n = 1037)** | **RIB + AI vs PAL + AI** | **ABE + AI vs PAL + AI** | **ABE + AI vs RIB + AI** |
| Age at mBC diagnosis, years | | | | | | |
| Mean (SD) | 65.1 (11.8) | 64.7 (12.0) | 64.8 (12.1) | -0.0324 | -0.0241 | 0.0081 |
| Median (IQR) | 66.0 (16.0) | 66.0 (17.0) | 66.0 (17.0) |  |  |  |
| Sex, *n* (%) | | | | | | |
| Male | 63 (1.1) | 12 (0.9) | 12 (1.1) | -0.0149 | 0.0049 | 0.0198 |
| Female | 5674 (98.9) | 1261 (99.1) | 1025 (98.9) |  |  |  |
| Race, *n* (%) | | | | | | |
| White | 3534 (61.6) | 783 (61.5) | 644 (62.1) | -0.0013 | 0.0093 | 0.0106 |
| Black | 551 (9.6) | 120 (9.4) | 97 (9.4) | -0.0065 | -0.0072 | -0.0007 |
| Other | 1652 (28.8) | 370 (29.0) | 296 (28.6) | 0.0056 | -0.0053 | -0.0109 |
| Practice type, *n* (%) | | | | | | |
| Community | 4877 (85.0) | 1090 (85.7) | 875 (84.4) | 0.0187 | -0.0175 | -0.0362 |
| Academic | 860 (15.0) | 182 (14.3) | 162 (15.6) |  |  |  |
| Insurance type, *n* (%) | | | | | | |
| Commercial health plan plus any other | 2084 (36.3) | 434 (34.1) | 355 (34.2) | -0.0463 | -0.0441 | 0.0022 |
| Commercial health plan | 1890 (32.9) | 470 (36.9) | 394 (38.0) | 0.0840 | 0.1052 | 0.0212 |
| Medicare | 250 (4.4) | 50 (3.9) | 30 (2.9) | -0.0205 | -0.0798 | -0.0595 |
| Medicaid | 112 (2.0) | 32 (2.5) | 13 (1.3) | 0.0386 | -0.0529 | -0.0908 |
| Other payer type | 1401 (24.4) | 286 (22.5) | 245 (23.7) | -0.0460 | -0.0178 | 0.0282 |
| Disease stage at initial diagnosis, *n* (%) | | | | | | |
| I | 609 (10.6) | 137 (10.8) | 112 (10.8) | 0.0045 | 0.0065 | 0.0020 |
| II | 1289 (22.5) | 287 (22.6) | 236 (22.7) | 0.0026 | 0.0059 | 0.0034 |
| III | 595 (10.4) | 133 (10.4) | 106 (10.2) | 0.0023 | -0.0056 | -0.0080 |
| IV | 2929 (51.1) | 645 (50.7) | 527 (50.8) | -0.0074 | -0.0055 | 0.0019 |
| Not documented | 315 (5.5) | 71 (5.5) | 57 (5.5) | 0.0023 | -0.0002 | -0.0024 |
| ECOG PS, *n* (%) | | | | | | |
| 0 | 2123 (37.0) | 474 (37.3) | 381 (36.7) | 0.0053 | -0.0057 | -0.0110 |
| 1 | 1535 (26.7) | 333 (26.1) | 279 (26.9) | -0.0136 | 0.0032 | 0.0168 |
| 2, 3, or 4 | 684 (11.9) | 153 (12.0) | 125 (12.0) | 0.0028 | 0.0024 | -0.0004 |
| Not documented | 1395 (24.3) | 313 (24.6) | 253 (24.4) | 0.0058 | 0.0013 | -0.0045 |
| Disease-free interval, *n* (%) | | | | | | |
| De novo mBC | 2929 (51.1) | 645 (50.7) | 527 (50.8) | -0.0074 | -0.0055 | 0.0019 |
| ≤ 1 year | 239 (4.2) | 53 (4.2) | 43 (4.2) | 0.0005 | 0.0001 | -0.0005 |
| > 1-5 years | 875 (15.2) | 197 (15.4) | 155 (15.0) | 0.0055 | -0.0074 | -0.0129 |
| > 5 years | 1694 (29.5) | 378 (29.7) | 312 (30.1) | 0.0035 | 0.0118 | 0.0083 |
| Visceral metastasis, *n* (%)^a^ | | | | | | |
| No | 3740 (65.2) | 828 (65.1) | 678 (65.4) | -0.0027 | 0.0047 | 0.0074 |
| Yes | 1997 (34.8) | 445 (34.9) | 359 (34.6) |  |  |  |
| Bone-only metastasis, *n* (%)^b^ | | | | | | |
| No | 3092 (53.9) | 686 (53.9) | 561 (54.1) | 0.0005 | 0.0048 | 0.0043 |
| Yes | 2646 (46.1) | 587 (46.1) | 476 (45.9) |  |  |  |
| Number of metastatic sites, *n* (%)^c^ | | | | | | |
| 1 | 3361 (58.6) | 744 (58.5) | 606 (58.4) | -0.0026 | -0.0026 | 0.0000 |
| 2 | 1338 (23.3) | 305 (23.9) | 242 (23.3) | 0.0148 | -0.0003 | -0.0151 |
| ≥ 3 | 517 (9.0) | 111 (8.7) | 93 (8.9) | -0.0105 | -0.0024 | 0.0081 |
| Not documented | 521 (9.1) | 113 (8.9) | 96 (9.3) | -0.0071 | 0.0073 | 0.0144 |
| Menopausal status at initial diagnosis, *n* (%) | | | | | | |
| Premenopausal | 1073 (18.7) | 277 (21.8) | 202 (19.5) | 0.0767 | 0.0205 | -0.0562 |
| Postmenopausal | 4305 (75.0) | 919 (72.2) | 763 (73.5) | -0.0637 | -0.0344 | 0.0294 |
| Not documented | 297 (5.2) | 64 (5.0) | 60 (5.8) | -0.0056 | 0.0282 | 0.0338 |
| Not applicable (patient is male) | 63 (1.1) | 12 (0.9) | 12 (1.1) | -0.0149 | 0.0049 | 0.0198 |
| Year of index date, *n* (%) | | | | | | |
| 2017 | 718 (12.5) | 69 (5.4) | 0 | -0.2509 | -0.5347 | -0.3379 |
| 2018 | 793 (13.8) | 122 (9.6) | 57 (5.5) | -0.1325 | -0.2852 | -0.1554 |
| 2019 | 870 (15.2) | 103 (8.1) | 126 (12.1) | -0.2220 | -0.0885 | 0.1342 |
| 2020 | 927 (16.2) | 92 (7.3) | 146 (14.1) | -0.2794 | -0.0586 | 0.2217 |
| 2021 | 1024 (17.9) | 80 (6.3) | 189 (18.2) | -0.3601 | 0.0089 | 0.3688 |
| 2022 | 869 (15.1) | 225 (17.6) | 245 (23.6) | 0.0678 | 0.2164 | 0.1486 |
| 2023 | 537 (9.4) | 582 (45.7) | 275 (26.5) | 0.8914 | 0.4583 | -0.4088 |
| Median follow-up duration (IQR), months | 31.3 (31.0) | 15.7 (20.8) | 21.4 (25.0) |  |  |  |

^a^ Visceral disease is defined as metastatic disease in the lung and/or liver; patients could have had other sites of metastases.
^b^ Bone-only disease is defined as metastatic disease in the bone only.
^c^ Multiple metastases at the same site were counted as one site (eg, 3 bone metastases in the spine was considered only one site).

Abbreviations: ABE, abemaciclib; AI, aromatase inhibitor; ECOG PS, Eastern Cooperative Oncology Group performance status; IQR, interquartile range; mBC, metastatic breast cancer; PAL, palbociclib; RIB, ribociclib; sIPTW, stabilized inverse probability of treatment weighting; SD, standard deviation.

Supplementary Figure S1. Overall survival in the (a) unadjusted analysis and (b) after sIPTW among the 3 CDK4/6 inhibitors in patients who started index treatment from 2017


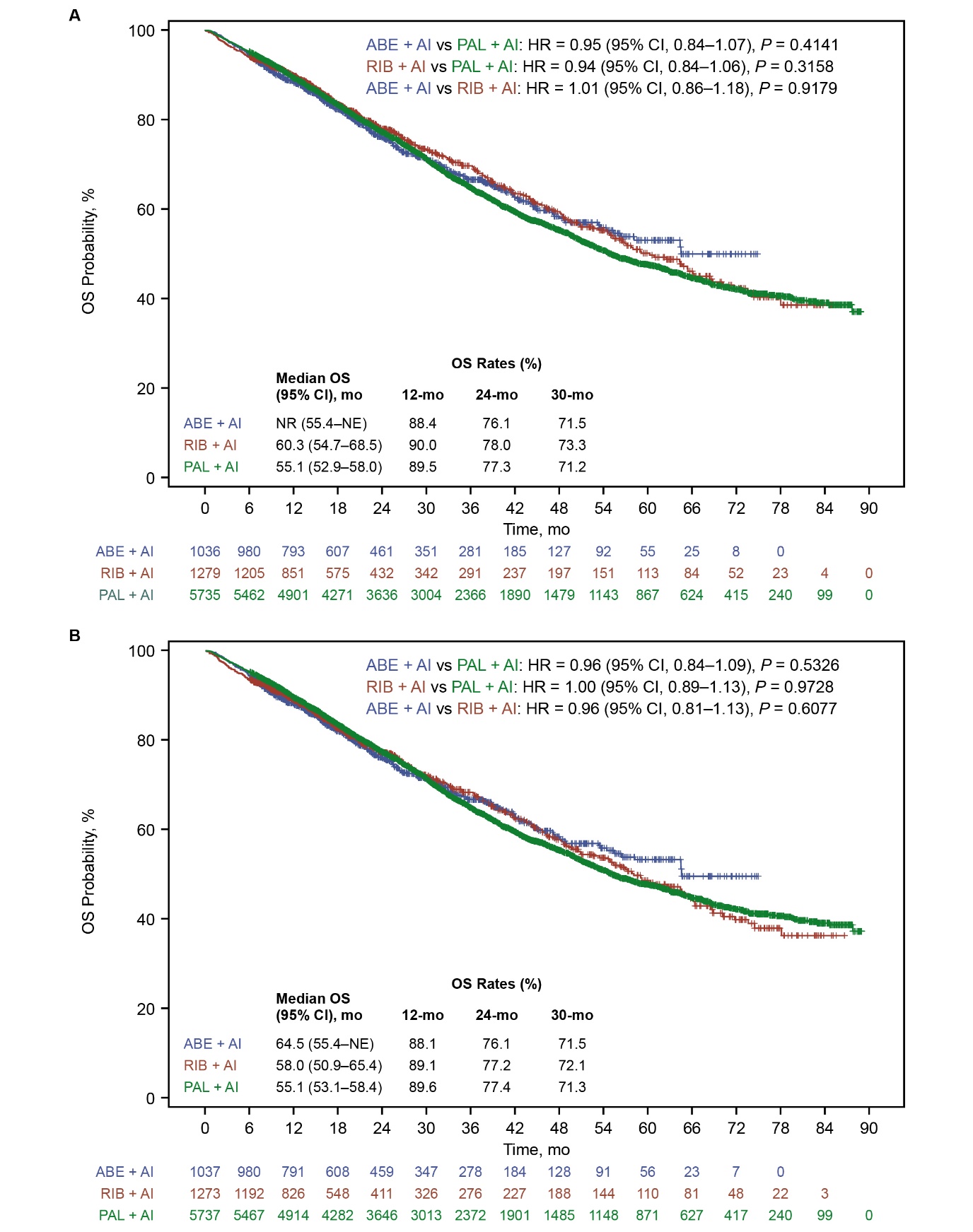


Abbreviations: ABE, abemaciclib; AI, aromatase inhibitor; CDK4/6, cyclin-dependent kinase 4/6; CI, confidence interval; HR, hazard ratio; mo, months; NE, not estimable; NR, not reached; OS, overall survival; PAL, palbociclib; RIB, ribociclib; sIPTW, stabilized inverse probability of treatment weighting.

Supplementary Figure S2. Forest plot of overall survival by subgroup after sIPTW in patients who started index treatment from 2017: (a) abemaciclib + AI vs palbociclib + AI, (b) ribociclib + AI vs palbociclib + AI, and (c) abemaciclib + AI vs ribociclib + AI


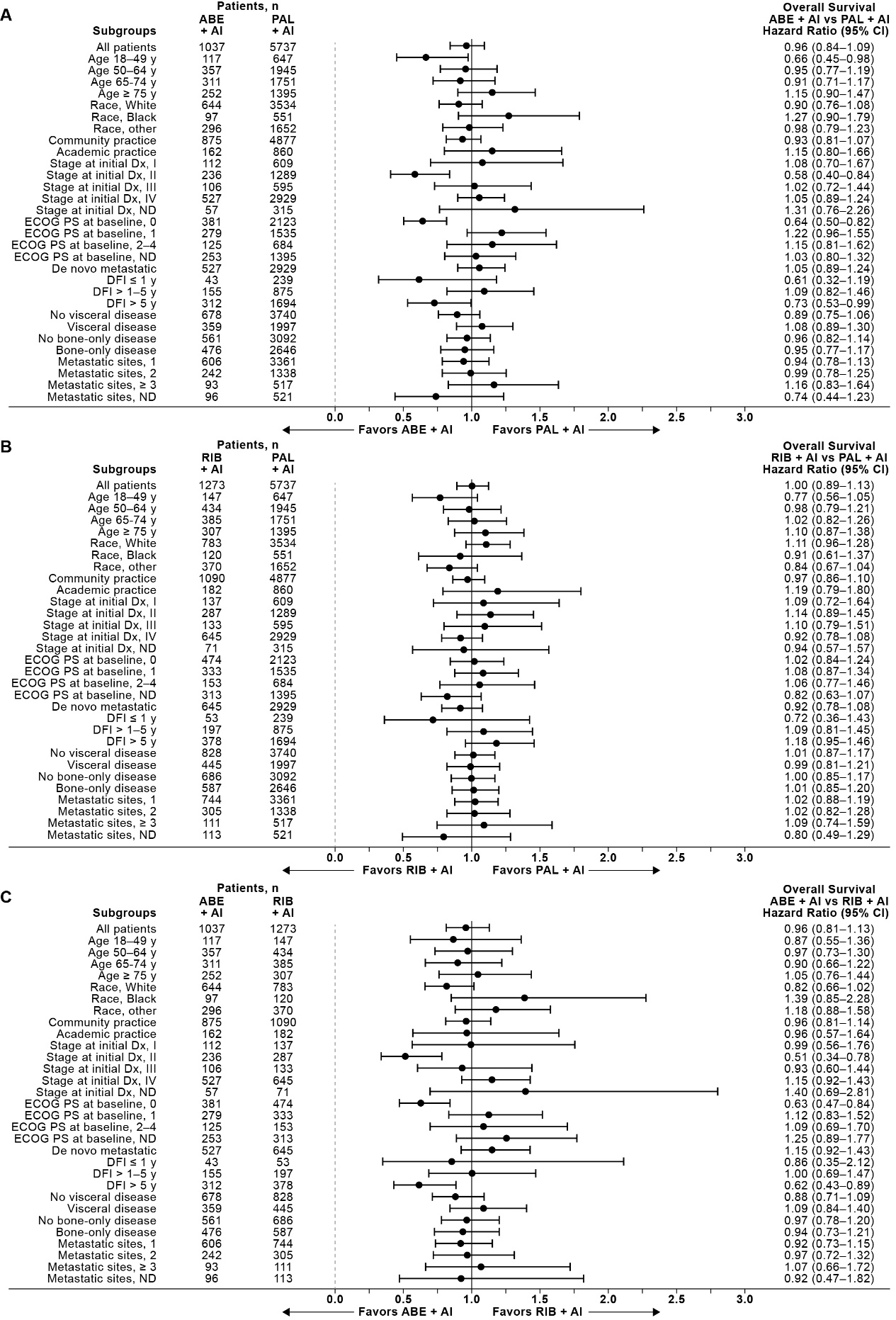

Abbreviations: ABE, abemaciclib; AI, aromatase inhibitor; CI, confidence interval; DFI, disease-free interval; Dx, diagnosis; ECOG PS, Eastern Cooperative Oncology Group performance status ND, not determined; PAL, palbociclib; RIB, ribociclib; sIPTW, stabilized inverse probability of treatment weighting; y, years.
